# Cambrian archaeocyathan metazoans: revision of morphological characters and standardization of genus descriptions to establish an online identification tool.

**DOI:** 10.3897/zookeys.150.1566

**Published:** 2011-11-28

**Authors:** Adeline Kerner, Françoise Debrenne, Régine Vignes-Lebbe

**Affiliations:** 1 CNRS (UMR 7207, centre de recherche sur la paléobiodiversité et les paléoenvironnements), Laboratoire Informatique et Systématique, MNHN Département Histoire de la Terre, Bâtiment de Géologie, CP48, 57 rue Cuvier, 75005 Paris (France); 2 13 rue du long foin, 91700 Sainte Geneviève des bois (France); 3 UPMC, université Pierre et Marie Curie, (UMR 7207, centre de recherche sur la paléobiodiversité et les paléoenvironnements), Laboratoire Informatique et Systématique, MNHN Département Histoire de la Terre, Bâtiment de Géologie, CP48, 57 rue Cuvier, 75005 Paris (France)

**Keywords:** Archaeocyatha, Cambrian, standardized characters, identification key, knowledge base, XPER²

## Abstract

Archaeocyatha represent the oldest calcified sponges and the first metazoans to build bioconstructions in association with calcimicrobes. They are a key group in biology, evolutionary studies, biostratigraphy, paleoecology and paleogeography of the early Cambrian times. The establishing of a new standardized terminology for archaeocyathans description has permitted the creation of the first knowledge base in English including descriptions of all archaeocyathan genera. This base, using the XPER² software package, is an integral part of the -Archaeocyatha- a knowledge base website, freely available at url http://www.infosyslab.fr/archaeocyatha. The website is composed of common information about Archaeocyatha, general remarks about the knowledge base, the description of the 307 genera recognized with images of type-specimens of type-species for each genus, as well as additional morphological data, an interactive free access key and its user guide.

The automatic analysis and comparison of the digitized descriptions have identified some genera with highly similar morphology. These results are a great help for future taxonomic revisions and suggest a number of possible synonymies that require further study.

## Introduction

Archaeocyathan represent the earliest reefal metazoan faunas dated at 521my, predating the Burgess Shale fauna and postdating Ediacarian faunas. They are exclusively Cambrian organisms that built the first metazoan bioconstructions as corals do today. Discovered in the middle of the XIXth century in the oldest fossiliferous rocks of Labrador, Canada, their geographical distribution is world-wide including Antarctica, Argentina, Australia, Canada, China, Germany, Greenland, France, Kazakhstan, Mongolia, Morocco, Poland, Uzbekistan, Sardinia, Serbia, South Africa, Spain, Russia and USA. Since their discovery, intensive studies have been carried out through international cooperation. Consensus about the phylogenetic relationships and biostratigraphic significance of these enigmatic organisms has been now achieved (see summary in Debrenne and Zhuravlev 1992).

As mysterious fossils without recent close-relatives, archaeocyathan represent an extinct class of the phylum Porifera, close to the Demospongiae (Debrenne and Vacelet 1984; Debrenne and Zhuravlev 1992). Their skeleton is commonly preserved as carbonate within limestone, which precludes their mechanical or chemical extraction from the surrounding matrix. Therefore, their complex, sometimes problematic morphology has to be examined through thin sections. As a consequence the orientation of the section through the skeleton, which influences the description and identification of the specimen is poorly controlled or even random. Identification of incomplete specimens is also highly problematic and the lack of specialists in the field aggravates this. As important Cambrian organisms, it is necessary for specialists and non-specialists to be able to rapidly and unambiguously identify specimens. However, easy to use identification keys are lacking despite several attempts to create such tools. Rozanov, using Vavilov’s Law (Vavilov 1922), produced tables with homologous series, used as identification systems (Rozanov and Missarzhevskiy 1966). The variability in the homologous series of Archaeocyatha contains three groups of primary skeletal elements: the outer wall, the inner wall and the intervallum. Identification can be obtained using combinations of these three groups of characters but this approch, which resembles an identification key, is still complex and inapplicable to incomplete specimens.

Archaeocyathan databases have been successively developed since the 1980s. These include a database on Ajacicyathida by Debrenne and Prieur (1981), a computer aided identification with single access key, called ECAD, by M. and F. Debrenne (never circulated and stopped due to ongoing taxonomic revisions (Debrenne et al. 2002)), and a first, French version of the XPER² knowledge base (Debrenne and Kerner 2006). All these databases, with the exception of the XPER² system, used a fixed sequence of character choices that is insufficiently flexible to fit the morphological complexity of the archaeocyathans. This paper is aimed at introducing (1) a new standardized terminology for archaeocyathans description applicable in knowledge base, (2) the first knowledge base including descriptions of all archaeocyathan genera with a free access identification key, and (3) some outputs of the dataset. A brief review of archaeocyathan anatomy, systematics and importance in the Cambrian system is first given. The results of this study are freely accessible on the Internet at: http://www.infosyslab.fr/archaeocyatha.

## Introduction to Archaeocyatha

### Anatomy and systematics of Archaeocyatha.

**Figures 1–3.**
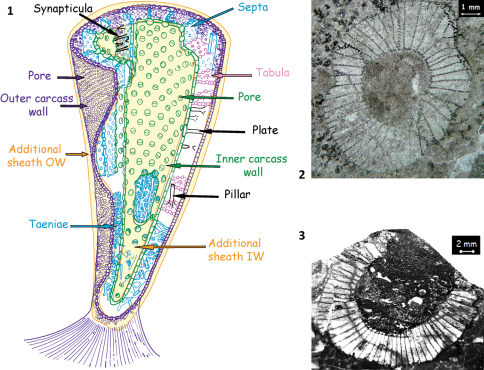
**1** Stylized archaeocyathan skeleton (Debrenne, 1964 modified) **2** *Erismacoscinus* sp. in transverse section. specimen 2474 4.2Tb MNHN, Paris collection Destombes, Jbel Taissa, Morocco **3** *Coscinocyathus dianthus* Bornemann, lectotype GML An597, Canal Grande, Sardinia (Bornemann 1886).

Morphologically, the archaeocyathan skeleton is composed of two inverted porous cones, fitting into each other and interpreted as outer and inner walls delimiting the intervallum. Vertical radial elements (septa, taeniae) and/or horizontal elements (tabulae) connect the two walls (Fig. 1). The archaeocyathan cups display various architectural types: one-walled conical, single-chambered subspherical, multi-chambered conical (thalamid), chaetetid, and syringoid with solitary or modular (pseudocolonial) habits. Their skeleton is primarily made of globally polyhedral crystallites of high-magnesian calcite, probably the result of an organic matrix mediated process at a very primitive stage.

Archaeocyathan systematics is based on skeletal ontogeny determining the order of appearance of skeletal elements, their degree of complication and the stabilization of adult features. Orders are characterized by the architecture of the cup, suborders by growth pattern models, superfamilies by the outer wall types, families by the inner wall types. Genera are differentiated by variations in walls and intervallar types, as well as distribution of pores in each element. Species are separated by different numerical coefficients (Debrenne et al. 1990; Debrenne and Zhuravlev 1992; Debrenne et al. 2002). The Class Archaeocyatha is composed of six orders and twelve suborders. The previous conventional subdivision into Regulares and Irregulares is often still used in biostratigraphy or paleoecology. These subdivisions roughly correspond to Ajacicyathida and Coscinocyathida (ex-Regulares) and Archaeocyathida and Kazachstanicyathida (ex-Irregulares).

### The role of archaeocyathan in the Cambrian System.

Archaeocyatha are of prime importance in biostratigraphic studies. The first stage subdivision based on archaeocyathan was established on the Siberian platform (Zhuravleva 1960). Subdivison of the Cambrian System was traditionally based on trilobite occurrences. However, the discovery of a rich archaeocyathan fauna on the Siberian Platform in horizons below the first appearance data (FAD) of trilobites, provided evidence for the establishment of a new stage, the Tommotian (Rozanov and Missarzhevskiy 1966), subdivided in 3 zones (Tab.1). Since then, archaeocyathan biozones have been used in key Cambrian areas such as Siberia, Morocco, Spain, Canada and Australia. The distribution of archaeocyathans in time, mainly early Cambrian with few relicts in middle and late Cambrian, limits their use to stages 2 to 4 of the International Stratigraphic Chart. Two parallel scales, one based on trilobites the other on archaeocyathan are established when possible for many Cambrian localities where archaeocyathan and trilobites are well studied. Under certain conditions, archaeocyathan may provide finer biozones than trilobites (Table 1).

**Table 1.** Comparison of archaeocyathan and trilobites biozones (modified after Rozanov and Sokolov 1984 and Mansy et al. 1993).

STAGES	SEBERIAN PLATFORM		ALTAI SAYAN		LAURENTIA	
	Archaeocyatha	Trilobita	Archaeocyatha	Trilobita	Archaeocyatha	Trilobita
Stage 4?Toyonian	*Irinaecyathus grandiperforatus*	*Anabaraspis splendens*	*Erbocyarhus heterovallum*	*Kooteniella-Edelsteinaspis*		*Plagiura* / *Poliella*
		*Lermontova grandis*	*Irinacyathus ratus*		*Tegerocyathus greenlandensis* / *Pycnoidocyathus pearylandicus*	
		*Bergerionella ketemensis*	*Adaecyathus solidus*	*Parapoliella*-*Oncocephalina*	*Archaeocyathus atlanticus*	
Stage 4?Botoman		*Bergerionaspis ornata*	*Syringocyathus aspectabilis*		*Pycnoidocyathus serratus* / *Tabulaconus kordae*	*Bonnia* / *Olenellus*
		*Bergerionellus asiaticus*	*Tercyathusaltaicus*	*Poliellina*-*Laticephalus*	*Claruscoscinus fritzi* / *Metacyathelluscaribouensis*	
		*Bergerionellus gurarii*				
	*Porocyathus squamosus, Botomaecyathus zelenovi*	*Bergerionellus micmacciformis* / *Erbiella*	*Clathricoscinus*		*Ethmophyllum whitneyi* / *Sekwicyathus nahaniensis*	
Stage 3?Atdabanian	*Fansycyathus lermontovae*	*Judomia*	*Arturocyathus torosus*	*Sajanaspis*-*Kameshkoviella*		"Nevadella"
			*Nalivkinicyathus cyroflexus*			
	*Nochoroicyathus kokoulini*					
	*Carinacyathus pinus*	*Pagetiellus anabarus*	*Thalamocyathus howelli*	*Resimopsis*		"Fallotaspides"
	*Retecoscinus zegebarti*		*Nochoroicyathus marinskii*			
		*Profallotaspis jakutensis*				
Stage 2?Tommotian	*Dokidocyathus lenaicus* / *Tumuliolynthus primigenius*					
	*Dokidocyathus regularis*					
	*Nochoroicyathus sunnaginicus*					
Fortunian						

Another interest concerns their paleoecology. Detailed studies of archaeocyathan settlements show that they were adapted to a narrow temperature range, corresponding to the intertropical zone. They were stenohaline organisms, living in the soft substrates of the intertidal zone. As passive filter-feeders, they are more adapted to habitats with reduced turbulence. Since Cambrian rocks lack usual climatic indicators such as tillites, modern phosphorites, or clays containing fossils of terrestrial vegetation, archaeocyathan with their retricted living conditions, are good indicators for ecological and environmental reconstructions (Debrenne et al. 2002; Gandin and Debrenne 2010). They are also significant in paleobiogeography. Reconstructions of land distribution are difficult for the Precambrian/Cambrian periods due to problems of paleomagnetism. Archaeocyathan reef distributions in epireic seas constrain map building. Five provinces are recognized after a first phase cluster analysis of generic distribution data: Siberia-Mongolia, Central East-Asia, Europe-Morocco, Australia-Antarctica, North-America-Koryakia. Two realms are defined by a second phase cluster analysis: Eurasia – the three first provinces – and Lauraustral – the last two provinces (Kruse and Shi 2000). Moreover, the pathways of archaeocyathan migrations inferred from the Jaccard Coefficient (Kruse and Shi 2000) confirm the early Cambrian existence of East and West Gondwana, the rifting of Laurentia from the Australian-Antarctic margin, and the drift of suspect Altay Sayan and Mongolia terrains of the Chinese East Gondwana margin towards Siberia (Debrenne et al. 1999). These results highlight the role of archaeocyathan as key group for fundamental problems in paleobiology and geology and the important support the exhaustive compendium of archaeocyathan species and genera, along with efficient identification key, may provide for future studies.

## Standardized terminology for Archaeocyatha

### Proposition for an adapted terminology.

A digitized knowledge base can be enhanced if the taxonomic descriptions can be compared through automatic processes. This is possible if descriptions are written using a common and standardized set of characters. However, in the literature, descriptions are often heterogeneous, using different terms or described according to a specialist’s interpretation. The task to standardize the descriptions of Archaeocyatha was easier thanks to series of recent systematic revisions (Debrenne et al. 1990; Debrenne and Zhuravlev 1992; Debrenne et al. 2002). Despite this, some problems appeared due to equal states. For example, the difference between an “arcuate” structure and a “curved” structure is not immediately clear. These states may be identical, but each potential equivalent term has to be checked carefully before synonomising to a single term in the knowledge base. Reconciling traditional morphological terms is necessary in character construction for databasing. Character standardization reveals some hidden problems due to diagnoses and terminology. A classical diagnosis often follows this pattern:

Outer wall + one complex descriptive term,

inner wall + one complex descriptive term,

type of radial structure +/- other intervallar structure (tabulae…)

With such a structure, vocabulary homogenization is not adequate. Most of the terms included several concepts. The standardization step here requires the subdivision of complex descriptive terms into a list of terms with only one notion included. For example, the term “cambroid pores” contains information about the shape and the repartition of pores. Each character and state should be examined from all aspects and only basic descriptors (composed of only one notion) should be retained. This new organization of descriptors means the appearance of new terms and the disappearance of some classical terms. Moreover, in monographs, diagnoses are built only with characters that have a taxonomic interest. Some states and/or descriptors do not have any taxonomic value but are highly visual and helpful for identification e.g. descriptors 31 & 51 in the online knowledge base.

The main difference between the traditional terminology referring to Archaeocyatha and one adapted to a knowledge base concerns the description of walls: terms have been dissected into basic descriptors and grouped differently (Table 2).

**Table 2.** Classical terminology referring to archaeocyathan walls and their equivalent basic descriptors.

outer wall and inner wall												
usual terms		new standardized terminology										dependent characters
		carcass wall						additionnal sheath				
		structure well defined	perforations	bumps		external plates	morphological tubes	present	micro-porous sheath	mesh	sieve	
				perforations	opening							
rudimentary		no	pores	none		none	none	no				description of types and repartition of pores
simple		yes	pores	none		none	none	no				
basic		yes	pores	none		none	none	no				
concentrical		yes	pores	none		none	none	no				
with canals		yes	canals	none		none	possible	no				description of canals
								yes	possible	none	possible	
						bracts, scales, unnuli	yes	no				
								yes	possible	none	possible	
with tumuli	simple	yes	pores	simple	downwarldy	none	none	no				description of bumps
	multiperforate			multiperforate	downwarldy							
pustular		yes	pores	simple	central	none	none	no				
with bracts scales or poretubes		yes	pores	none		yes	none	no				description of external plates
								yes	yes			
			canals	none		yes	yes	no				
								yes	possible		possible	
with annuli		yes	pores	none		yes	possible	no				
								yes	yes			
			canals	none		yes	possible	no				
								yes	yes			
compound		yes	pores	none		possible	none	yes	possible	none	possible	decriprion of additional sheath and of carcass structures
with microporous sheath		yes	pores	none		possible	none	yes	yes	none	none	
tellelar		yes	pores	none		none	none	yes	none	yes	none	
clathrate		yes	pores	none		none	none	yes	none	yes	none	
pseudoclathate		yes	pores	none		none	none	yes	none	yes	none	
tabular		link to tabulae descriptors: tabulae are extensions of inner wall, of outer wall or of both wall										

We consider that a wall can be composed of one or two parts. The first one, always present, is named a carcass wall (descriptors 6, 7, 11 to 28 & 31 to 50) and the second is an additional wall (descriptor 52 to 63). A carcass wall generally has perforations (pores or canals) (descriptors 11 to 21 & 31 to 44) and may have different structures: bumps (tumuli, putulae) (descriptors 22 to 24) or external plates (spines, bracts, scales, annuli) (descriptors 25 to 28 & 45 to 50). Additional walls group together the microporous sheaths (descriptors 53 to 55, 57 to 60 & 63), sieves formed by protrusions (compound walls: incipient pore subdivision and completely subdivided pores) (descriptors 53, 55 to 58, 60, 62 & 63) and mesh (tabella, clathri, pseudoclathri) (descriptors 58 & 61). Each element is described inside these new associations. This new organization included all usual wall types apart from tabular walls that are considered to be linked to tabulae (descriptor 80). For example, a simple tabular outer wall is considered as a single character in traditional terminology, here it is decomposed into different components: outer wall is in one part (no additional sheath), (descriptor 52), this part is composed of simple pores (descriptors 11 & 12) and, moreover, in the intervallum there are tabulae (descriptor 79) stemming from the outer wall curve line (descriptor 80).

Tabulae have been subdivided into two descriptors. The first one describes their construction (descriptor 80): independent of both walls (simple, pectinate, plate and membrane tabulae) or dependent on the inner wall, the outer wall or both walls (curved, simple segmented, concentric segmented and compound segmented tabulae). The second one describes the porosity of tabulae (descriptor 81).

### Modifications of the traditional terminology.

Different causes can justify the modification of used terminology: a single term refers to two or several different structures, two different terms refer to different things but introduce confusion between two different structures. The first example concerns the term “spines” that was used for two different structures: 1) external plates that look like bracts and 2) skeletal elements that divided pores to form an additional sheath. In the first case the term “spines” is retained whereas the second now corresponds to “protrusions”. The second example concerns sub-spherical chambered canals that may easily be confused with what we refer to as “chamber”, hence our preference for the term “curved canals”. However, the terminology referring to communicating canals appears difficult to understand for novices. We have chosen non porous, porous and spongiose to replace non-communicating, simple communicating and anastomosing. Finally, the difference between completely subdivided and incipient pore subdivision appears only in additional sheaths with the descriptor 62 “type of sieves”. Both are considered subdivided in the description of carcass wall pores (descriptors 12 & 32).

The second instance of terms that necessitated modification concern updating the character states. The Checkbase function in XPER² shows that the *Taylorcyathus* and *Connanulofungia* descriptions are similar and that new observations of their inner wall annuli show that they are stacked differently. A new state has therefore been created inside the knowledge base to describe the annuli of *Connanulofungia*: cone in cone (descriptor 49).

Other terms illustrating complex characters become useless after their division into basic descriptors. A first case concerns tumuli and pustulae. Simple tumuli and pustulae definitions are close together: these form bumps on a carcass outer wall and have a single opening, with a difference in the direction of the opening. With basic descriptors, a bump is described with its perforations oriented to the opening direction and the terms “tumuli” and “pustulae” presence therefore become redundant and inadequate (descriptors 22 to 24). The second case is about cambroid pores and anthoid pores. Pores are defined with some basic descriptors: their type (or shape), their distribution, their arrangement (descriptors 11 to 17 & 31 to 34). Cambroid pores are simple or polygonal pores (descriptor 12) with a regular distribution on the outer wall (descriptor 15) and a random arrangement (descriptor 16). Anthoid pores are polygonal pores (descriptor 12) with an irregular distribution (descriptor 15). With such deconstruction, anthoid pores and cambroid are not considered as terms to include in the knowledge base. In the case of basic and rudimentary walls these are quite difficult to distinguish. The term “rudimentary wall” is used for imperforate walls and for a skeleton without a carcass wall well defined: Tips of intervallar structures serve as carcass, and spaces between intervallar structures as carcass pores. The term “basic wall” means a carcass wall built with tips of intervallar structures too but with additional lintels between these, forming the carcass. Imperforate walls are defined as carcass walls that are well defined without perforations. The term “rudimentary perforate wall” is decomposed into carcass not well defined (descriptors 6 & 7), carcass pores irregular (descriptor 12 & 32), irregular repartition (descriptor 15) and one row of pores per intersept (descriptor 17 & 33) and basic wall into carcass well defined (descriptors 6 & 7), carcass pores irregular (descriptor 12 & 32), irregular repartition (descriptor 15) and 2 or more than 2 rows of pores per intersept (descriptor 17 & 33). The last discarded term is pseudotaeniae, defined as “taeniae with synapticulae at each interpore node”. In the knowledge base, this results in a descriptor association: vertical intervallar structures are taeniae (descriptor 65 & 66) and links are synapticulae which repartition is at each interpore node (descriptor 70 & 71).

## On line service for Archaeocyatha recognition

### Archaeocyathan knowledge base.

This was developed with Xper², which is software, available for use under a Creative Commons by-nc-nd license. The software is dedicated to storing structured descriptive data and to provide free (matrix) access keys (http://www.infosyslab.fr/lis/?q=en/resources/software/xper2, Ung et al. 2010).

The archaeocyathan knowledge base (Kerner et al. 2011) is composed of a set of standardized descriptions, one for each genus: all the descriptions use the same set of descriptors and character states. Terminology is therefore controlled and further documented by text and images. Images each have their own copyrights. The dataset (without images) is distributed for use under a Creative Commons license (by-nc-nd, see http://creativecommons.org/licenses/by-nc-nd/3.0/).

Most of characters proposed by paleontologists to identify archaeocyathan genera were collected from relevant literature (Debrenne et al. 1990; Debrenne and Zhuravlev 1992, Debrenne et al. 2002) and confirmed by observation of about 1000 specimens in the collections of the Museum National d’Histoire Naturelle, which contain about 600 type and illustrated specimens.

The knowledge base is composed of 307 genera considered to be valid at present with a world-wide geographical coverage. Stratigraphically, the knowledge base contains all the Cambrian deposits despite the predominance of Archaeocyatha in early Cambrian deposits. Each genus is illustrated with type specimens of the type species and some additional specimens. A total of 120 descriptors are used, 85 corresponding to morphological and ontogenetic data, 8 to stratigraphic and geographic data and 27 refer to traditional classification data. To each descriptor and character state we associated a definition and images, and/or drawings.

### Free access key.

**Figure 4.**
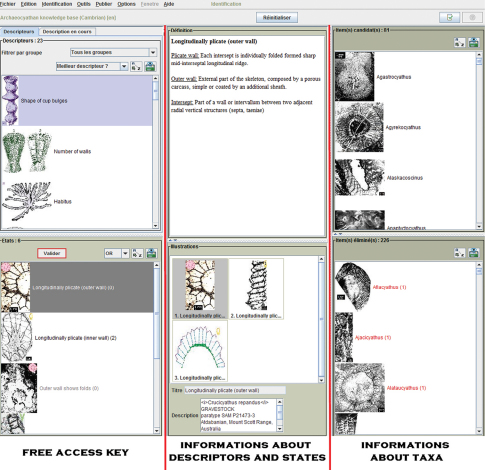
Screen shot of the free access key constructed in this study for archaeocyathan genera.

Incomplete specimens cannot be identified with traditional tools. Single access keys and natural keys (following the classification) are insufficiently flexible as they contain a predefined sequence of steps in the identification that rely on the presence of these distinguishing characters in the specimen. For example, if the outer walls (carcass more or less additional sheath) are not preserved, identification can only be made to suborder level. The identification service offered by Xper² is available offline or via the Internet as a free access key (Fig. 4). With free access keys (Hagedorn et al. 2010), the selection of a particular descriptor is chosen by the user at each step of the identification. The computer-aided identification tool deduces the remaining and eliminated taxa for each selected character and can display the reasons for each elimination (for various examples see Nimis and Vignes-Lebbe 2010). This type of identification system is very flexible and allows the identification of a specimen even if some of the described characters are not available, as is regularly the case for palaeontological specimens that are often incomplete. Moreover, the user can express doubt by selecting more than one character state, or chose another descriptor. Furthermore, at any step in the identification, the user can question why a taxon should have been eliminated and can check the descriptors that are incompatible with the specimen description. Another advantage of free access keys concerns superficially similar genera that do not belong to the same Order or Suborder. For example *Coscinocyathus* and *Erismacoscinus* are two genera that look very similar in transverse section even though they are not in the same Order (Fig. 2-3). *Coscinocyathus* has chambers, which is why it belongs to Capsulocyathida whereas *Erismacoscinus* does not have chambers and belongs to Ajacicyathida. The problem is that chambers are not visible in transverse section, so confusion is frequent. With free access keys, it is possible to identify these genera without using characters of the chamber. 

### Outputs and analysis of the knowledge base.

**Figure 5.**
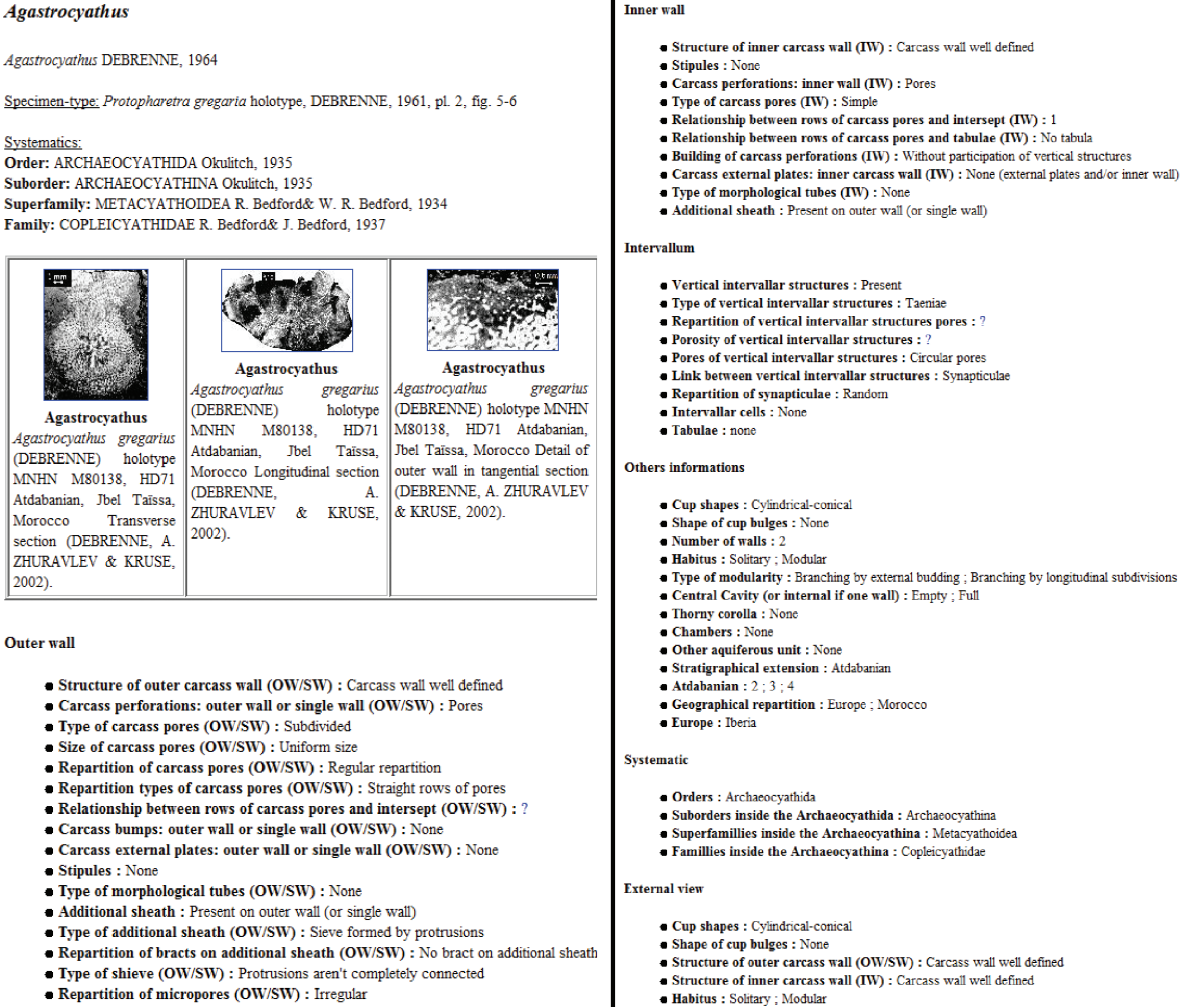
Agastrocyathus detailed sheet.

A complete form for each genus including descriptions, pictures and information concerning their systematics can be published from the system (Fig. 5).

**Figures 6–7.**
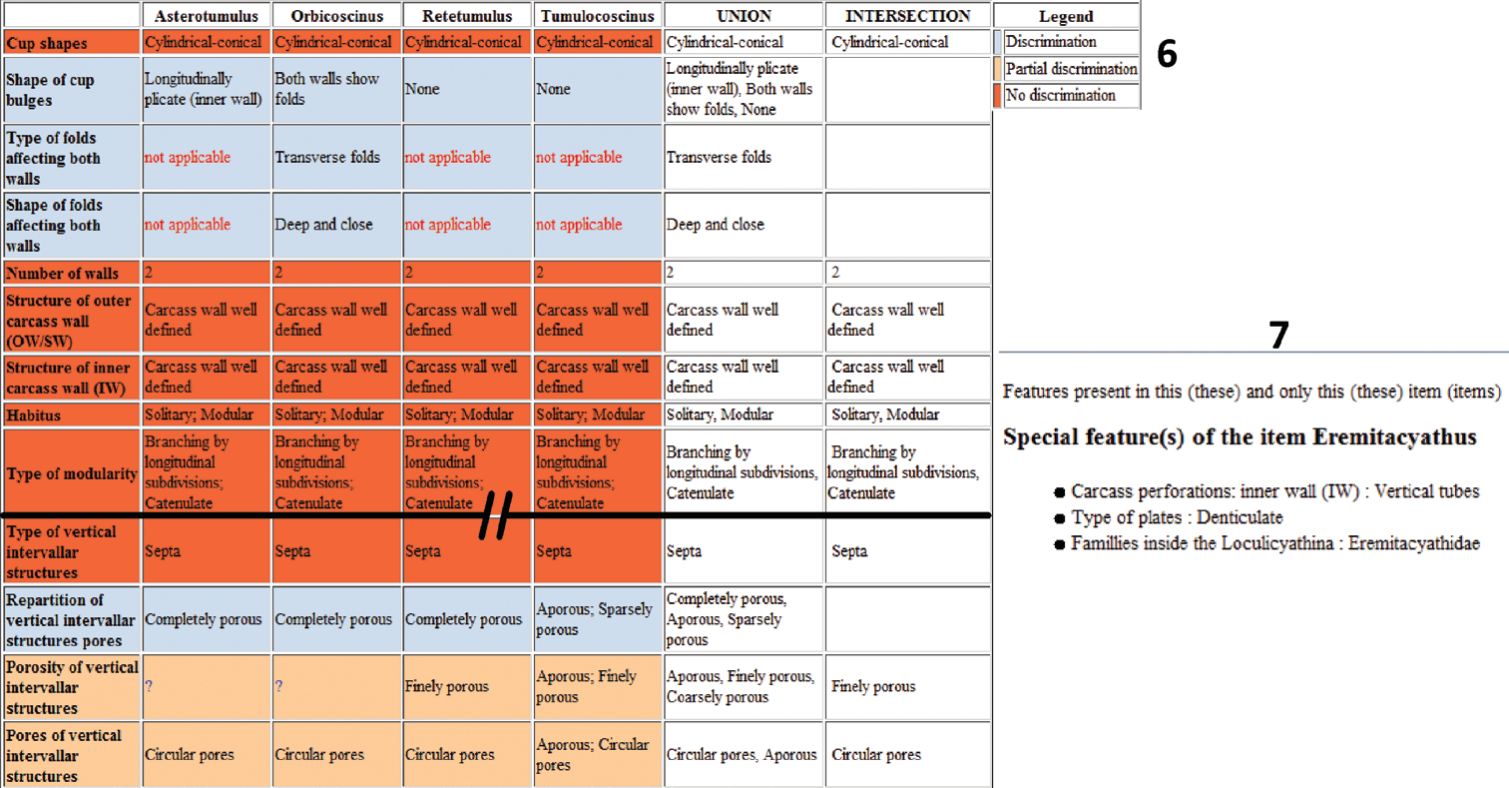
**6** Comparative table of Family Tumulocoscinidea **7** Special features of Eremitacyathus.

The Checkbase function compares all the pairs of taxonomic descriptions to see if they are distinguishable or if they overlap. If these conditions occur it means that some morphological aspects are compatible with more than one taxon. This iterative process is useful to check the consistency or misinterpretations of characters and the completeness of the knowledge base. It detected some similarities between *Graphoscyphia*, *Dictyocyathus* and *Molybdocyathus*. The three genera have an inner carcass wall with one row of simple pores per intersept and a dictyonal network. The only difference concerns the outer carcass wall which is basic or rudimentary. In recent literature, *Graphoscyphia* and *Dictyocyathus* have the same description: a basic outer carcass wall whereas *Molybdocyathus* has a rudimentary one. A fresh look at the specimens reveals that *Graphoscyphia* has a basic outer carcass wall (as originally described), *Dictyocyathus* does not have a basic outer carcass wall, but a rudimentary one and *Molybdocyathus* has a rudimentary one too (as originally described). With the change in the interpretation of the carcass wall structure of *Dictyocyathus*, the genus *Dictyocyathus* appears identical to *Molybdocyathus*. *Molybdocyathus* is now considered to be a junior synonym of *Dictyocyathus*.The automatic comparison of descriptions is displayed in a table, using different colors to highlight characters that are common or different in two or more genera. In Figure 6, we use this feature to visualize the morphological forms in the Tumulocoscinidea family. This tool has different uses. First, it can help to rapidly complete an identification when few taxa remain and differences can easily be seen. It can also be useful as a teaching tool for archaeocyathan identification. In the same way during the identification process, the information as to why a taxon is discarded (states incompatible are colored in red in the complete form of the discarded taxon) can be used as an efficient method to help the user with recognizing character states and descriptive terms.

Xper² can extract “special features” (Fig. 7), i.e. unique states present only in a single taxon. We used this feature here to check the data. It could also be used to help weight characters when creating a classical polytomous key. Some software already exists to create keys from data matrices, and in a near future we will connect our application to the webservices of the ViBRANT project (http://vbrant.eu/) to facilitate this.

### Archaeocyatha Website.

The archaeocyathan knowledge base and outputs are included inside a website about Archaeocyatha. This site is composed of different information types. The first part, called Archaeocyatha brings together common information about Archaeocyatha: an introduction, covering their role in Cambrian systems, their morphology and a bibliography. The second part is about the knowledge base. This is composed of general remarks about the knowledge base and some data exports from the system: list of genera and their detailed sheets, list of descriptors, list of groups of descriptors and the base properties. The last part concerns the interactive key and its tools: user guide, matching terminologies and glossary. Matching terminologies correspond to the list of all usual terms used in archaeocyathan descriptions. From this, the user can find how a traditional term appears in the knowledge base.

**Figure 8.**
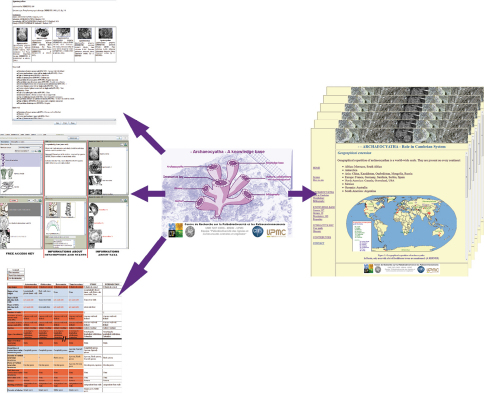
Archaeocyatha website structure. On the left, interactive key and some applications of it. On the right, an example of web page.

The new and complete English version of the archaeocyathan knowledge base (Cambrian) can be accessed at http://www.infosyslab.fr/archaeocyatha, and be used to identify an archaeocyathan specimen to generic level (Fig. 8).

## Conclusion

The identification of the Cambrian and predominantly early Cambrian metazoans referred to as Archaeocyatha, are important for a number of disciplines including biostratigraphy, paleoecology and paleogeography. Since the study of their morphological 3D-structures is complex due to different views in thin section, their identification is difficult. This problem is exacerbated by the lack of specialists in this field, with most now retired or involved in other projects. Establishing of a knowledge base for these organisms is a necessary tool and a first step to identify new field discoveries so that they can be placed in a wider context. The Xper² application for archaeocyathan genera is the first digitized content, in English, enabling identification with free access keys, and includes all currently accepted genera as well as illustrations of their nomenclatural types. A first version of the archaeocyathan knowledge base (Cambrian) is freely accessible online at URL http://www.infosyslab.fr/archaeocyatha. We hope that such an application constitutes an efficient resource for any further studies on Archaeocyatha.

The application is the first step of a general review on Archaeocyatha using the new tools for taxonomy. It will be completed and up-dated on an ongoing basis to follow and include new findings on these fossils. Content will focus on further characters analysis, both to refine the descriptions for paleontological studies, and to compute multidimensional characters. Tools will be developed to support further data analysis tool for discovering new discriminating characters. We plan to shift from a simple website (web 1.0) to a collaborative website (using Scratchpads see http://scratchpads.eu/) to open the application to the community of specialists and non specialists interested by Archaeocyatha data.
